# Fetal Alcohol Exposure Reduces Dopamine Receptor D2 and Increases Pituitary Weight and Prolactin Production via Epigenetic Mechanisms

**DOI:** 10.1371/journal.pone.0140699

**Published:** 2015-10-28

**Authors:** Omkaram Gangisetty, Olivia Wynne, Shaima Jabbar, Cara Nasello, Dipak K. Sarkar

**Affiliations:** 1 Endocrine Program, Department of Animal Sciences, Rutgers, The State University of New Jersey, New Brunswick, New Jersey 08901, United States of America; 2 Department of Genetics, Rutgers, The State University of New Jersey, New Brunswick, New Jersey 08901, United States of America; University of Queensland, AUSTRALIA

## Abstract

Recent evidence indicated that alcohol exposure during the fetal period increases the susceptibility to tumor development in mammary and prostate tissues. Whether fetal alcohol exposure increases the susceptibility to prolactin-producing tumor (prolactinoma) development in the pituitary was studied by employing the animal model of estradiol-induced prolactinomas in Fischer 344 female rats. We employed an animal model of fetal alcohol exposure that simulates binge alcohol drinking during the first two trimesters of human pregnancy and involves feeding pregnant rats with a liquid diet containing 6.7% alcohol during gestational day 7 to day 21. Control rats were pair-fed with isocaloric liquid diet or fed *ad libitum* with rat chow diet. Adult alcohol exposed and control female offspring rats were used in this study on the day of estrus or after estrogen treatment. Results show that fetal alcohol-exposed rats had increased levels of pituitary weight, pituitary prolactin (PRL) protein and mRNA, and plasma PRL. However, these rats show decreased pituitary levels of dopamine D2 receptor (D2R) mRNA and protein and increased pituitary levels of D2R promoter methylation. Also, they show elevated pituitary mRNA levels of DNA methylating genes (DNMT1, DNMT3b, MeCP2) and histone modifying genes (HDAC2, HDAC4, G9a). When fetal alcohol exposed rats were treated neonatally with a DNA methylation inhibitor 5-Aza deoxycytidine and/or a HDAC inhibitor trichostatin-A their pituitary D2R mRNA, pituitary weights and plasma PRL levels were normalized. These data suggest that fetal alcohol exposure programs the pituitary to increase the susceptibility to the development of prolactinomas possibly by enhancing the methylation of the D2R gene promoter and repressing the synthesis and control of D2R on PRL-producing cells.

## Introduction

A rapidly accumulating body of evidence indicates that many diseases must be understood in a life-long perspective, as trajectories that start at before or during conception and surface upon clinical detection decades later. Fetal alcohol exposure (FAE) occurs to the fetus when pregnant women drink alcohol, and causes a pattern of mental and physical defects collectively known as fetal alcohol spectrum disorders (FASD) [1]. The prevalence of FASD is estimated to be 1 in every 100 live births, and it is the leading cause of mental retardation in the western world [2]. In addition to damage in the central nervous system, FAE also causes hyperactive responses to stress challenges [3–5] and decreases body immune functions including lower T lymphocytes proliferation and function and NK cells cytolytic activity and increased incidence of bacterial infections [6–10]. Since the neuroendocrine-immune system is critically involved in the regulation of tumor surveillance, we have initiated studies to determine whether *in utero* alcohol exposure increases the offspring susceptiblity to tumorigenesis during adult life using rats as an animal model. We have recently reported that fetal alcohol exposure enhances the development of prostate carcinoma upon administration of N-nitroso-N-methylurea (NMU) with or without testosterone as carcinogens in adult offspring rats [11, 12]. However, the sensitivity to develop non-carcinogen induced tumors, like prolactin (PRL)-secreting pituitary adenomas (prolactinomas), in these animals has not been well studied.

Prolactinomas are the most common pituitary tumors in humans [13]. Several reports provided evidence that alcoholic men and women have higher incidence of prolactinomas [14–15]. Chronic alcohol consumption increases PRL levels in blood and cell proliferation in pituitary PRL-producing cells known as lactotropes, resulting in hyperprolactinemia in human and animals. [14–17] The molecular mechanism by which alcohol increases PRL secretion and lactotroph proliferation may involve dopamine receptors inhibitory control of lactotropic cell function [17–19]. Dopamine, a neurotransmitter secreted from the hypothalamus is the major inhibitor of PRL production and secretion, as well as lactotropes proliferation in pituitary gland [20–22]. Dopamine’s inhibitory effect on PRL production is mediated through the dopamine D2 receptor (D2R) [23]. D2R is the predominant dopamine receptor subtype in anterior pituitary and mediates dopamine’s inhibitory function in lactotropes [24]. D2R knock out mice display hyperprolactinemia and lactotrope hyperplasia [25, 26]. Studies also showed an evidence for an inhibitory effect of alcohol on the dopaminergic system [27] and D2R in the brain [28]. Estrogen, a natural hormone, when elevated for a prolonged period of time induces prolactinomas in various strains of rats including Fisher 344 strain [29]. It is also known to reduce dopamine levels and the D2R activity in lactotropes [30, 31]. Whether, fetal alcohol exposure programmed the pituitary to increase the susceptibility to the development of prolactinomas by repressing D2R production has not previously been tested. Epigenetic alterations such as promoter methylation has been shown to be a potential mechanism to regulate gene expression in fetal alcohol exposed rat offspring [32–34]. We hypothesized that fetal alcohol exposure epigenetically programs the D2R promoter methylation in the pituitary that lead to repression of the D2R expression and function in pituitary lactotropic cells resulting in the increased growth of the pituitary and secretion of PRL. We report that fetal alcohol exposure increases methylation of D2R proximal promoter and expression of DNA methyation genes while it decreases the expression of D2R and enhances tissue growth and production of PRL in the pituitary. We also show that treatment with DNA methylation blockers reverse fetal alcohol effects on D2R gene, PRL hypersecretion and pituitary growth in rats.

## Methods

### Animals

Fisher-344 strain rats were obtained from Harlan Laboratories (Indianapolis, IN), housed in a controlled condition of 12h light/dark cycle at a constant temperature 22°C throughout the study. Rats were housed in a group of two animals per Open-type Shoe Box Cages with Bedcob bedding, and they were fed with ad libitum rat chow and tap water in a conventional facility. Health statuses of animals were checked regularly by determining body weight, feeding and general behaviors, and university veterinarians were consulted to address any special health needs. Some of these rats were bred, and on gestational day 7 through 21, and they were fed either rat chow ad libitum (AD), a liquid diet containing ethanol (AF; Bioserve, Frenchtown NJ) ad libitum or pair-fed (PF; Bioserve) an isocaloric liquid control diet (with ethanol calories replaced by maltose-dextrin). The concentration of ethanol varied in the diet for the first 4 days from 1.7 to 5.0% v/v to habituate the animals with the alcohol diet. After this habituation period, animals were fed the liquid diet containing ethanol at a concentration of 6.7% v/v. Previous studies have shown that the peak blood ethanol concentration is achieved in the range of 120–150 mg/dl (0.12–0.15%) in pregnant dams fed with this liquid diet [35]. At postnatal day 2 (PD2), AF and PF pups were cross-fostered to untreated lactating AD dams to prevent any compromised nurturing by the AF and PF moms. Litter size was maintained at 8 pups per dam to minimize any nurturing effect on the body growth. Pups were weaned on PD21, and housed by sex. A total of 6–8 animals (n = 6–8) were used in each treatment group, by taking one offspring from each litter to avoid any gene homogeneity. Female offspring rats were used throughout the study.

In the first series of experiments, we determined whether fetal alcohol feeding changes pituitary lactotropic cell functions during adulthood. For this, a group of thirty-six AD, PF or AF female offspring rats at 90 days of age on the day of estrus were sacrificed by decapitation (6 to 8 animals/feeding group). Pituitary tissues and trunk blood samples were collected for weight measurement and prolactin (PRL) hormone assay. We then determined the effect of fetal alcohol exposure on estrogen-induced changes in PRL producing lactotropic cell functions. For this, a group of forty-eight animals at 60 days of age were ovariectomized under sodium pentobarbital anesthesia (50–60 mg/kg, i.p.) as a general anesthesia and 25% Bupivacaine (sc) as a local anesthesia and then subcutaneously implanted with an estradiol-17β (Sigma, St. Louis, MO) filled 1-cm silastic capsule (Dow Corning, Midland, MI) (8 animals/feeding group) or an empty 1-cm silastic capsule (8 animals/feeding group). After surgery, animals were kept under observation for pain and suffering or infection for 3 days. Estrogen-treated rats were kept in a cage lit with HEPA filter to protect user from estrogen contamination from this animals. These estradiol capsules were shown to maintain plasma levels of estradiol-17β between 120 and 150 pg/ml and induce prolactinomas [36]. We determined the time-response of estradiol effects on pituitary weight and PRL production. A group of rats were sacrificed by decapitation on 15 days after the estradiol implants and their pituitary tissues were collected to determine weight changes and lactotropic cell proliferation and trunk blood samples were obtained to measure plasma levels of PRL. We also employed forty-eight AD, PF or AF offspring rats treated with estradiol capsule for 60 (8 rats/group) or 90 days (8 rats/group) to collect trunk blood samples for measurements of plasma PRL and pituitary tissues for measurements of various genes. Pituitary levels of PRL, D2R and epigenetic genes were measured by real-time PCR (RT-PCR) assays, D2R gene methylation levels were measured by methylation specific PCR and pyrosequencing assay, and PRL and D2R protein levels by western blot measurements. In addition, a group of seventy-two AD, PF or AF offspring rats received subcutaneous injection of HDAC inhibitor Trichostatin-A (TSA; 2mg/kg Sigma; 6 rats/group), DNA methylation inhibitor 5-azadeoxy cytidine (5-AZAdC; 5mg/kg Sigma; 6 rats/group), TSA and 5-AZAdC (6 rats/group) or vehicle (6 rats/group) on day 2, 4 and 6 after birth in their home cage. After 60 days of birth they were ovariectomised and implanted with an estrogen capsule as described above. These rats were sacrificed by decapitation on 60 days after the implants and trunk blood samples and pituitary glands were collected and used for plasma PRL, pituitary weight and pituitary D2R gene expression and protein levels. Animal surgery and care were performed in accordance with institutional guidelines and complied with NIH policy. Rutgers Institutional Animal Care and Use Committee (IACUC) approve this research protocol.

### Lactotropic cell proliferation

At the end of experiment, some animals were intraperitoneally injected with bromodeoxyuridine (BrdU) (50 mg/kg body weight) 4 h prior to sacrifice. After sacrifice, the anterior pituitary were removed, weighed, fixed in 4% buffered formaldehyde, and used for immunocytochemical detection of BrdU-labeled lactotropes undergoing replicative DNA synthesis [36]. BrdU-labeling was done by employing BrdU monoclonal mouse IgG (1:200; Becton Dickinson Biosciences, San Jose, CA), while PRL labeling was done by using rabbit anti-rPRL (PRL-S9; 1:300,000, NHPP, Torrance, CA). At least 5,000 labeled and unlabeled cells from each anterior pituitary were counted.

### PRL hormone enzyme immunoassay (EIA)

Plasma PRL levels were measured using rat PRL EIA kit (Alpco Diagnostics, Salem, NH) as per the instructions from the manufacturer.

### Real time PCR for gene expression measurements

Gene expression levels of PRL, D2R, DNMT1, 3A, 3B, MeCP2, HDAC2, 4, G9A and SET7 in rat pituitaries were measured by quantitative real time PCR (SYBR green assay). Total RNA from pituitary gland was extracted using the All in One Purification Kit (Norgen Biotek, Ontario, Canada). Total RNA (1μg) was converted to first strand complementary DNA (cDNA) using high capacity cDNA reverse transcription kit (Applied Biosystems, Carlsbad, CA). All the primer sequences used for the study are given in Table 1. Real time quantitative PCR was performed at 95°C for 5 min followed by 40 cycles of 95°C for 15 sec, 60°C for 30 sec, 72°C for 40 sec in Applied Biosystems 7500 Real time PCR system. The quantity of target gene expression was measured using standard curve method. GAPDH, 18S RNA and ribosomal protein 19 (RPL-19) were used as internal controls.

**Table 1:** Primer sequences.

Primer Name	Sequence
PRL FP	5’ CAGAAAGTCCCTCCGGAACTT 3’
PRL RP	5’ AGGAGCTTCATGGATTCCACC 3’
D2R FP	5’ CCCAGAGAGGACCCGGTATAG 3’
D2R RP	5’ CTGGTTTGGCAGGACTGTCA 3’
D2RMF	5’ TAAAGGAGGGGATCGATTC 3’
D2RMR	5’ CTACTCGCCGATATCATCCT 3’
D2RUF	5’ TTTTAAAGGAGGGGATTGATTT 3’
D2RUR	5’ AAACTACTCACCAATATCATCCT 3’
D2RBSPFP	5’ TTATTTTTTTTTTAAAGATTTGATGT 3’
D2RBSPRP	5’-/5Bio/CCCCAAAACTATCTCTACACTA 3’
D2RseqFP1	5’ GATTTTTTAAAGGAGGG 3’
D2RseqFP2	5’ ATTTAAAAAGTATAGGAT 3’
DNMT1 FP	5’ CAAAGCAAGTGCAATCCCAAA3’
DNMT1 RP	5’ TACTTCAGGTCAGGGTCATCTAGGT 3’
DNMT3A FP	5’ TTCTTGAGTCTAACCCCGTGATG 3’
DNMT3A RP	5’ TGTTCATGCCAGGAAGGTTACC 3’
DNMT3B FP	5’ CCAAGTTGTACCCAGCGATTC 3’
DNMT3B RP	5’ CCACTTTAATACCCAAGTCCTTGAG 3’
MeCP2 FP	5’ CAAACAGCGACGTTCCATCA 3’
MeCP2 RP	5’ TGTTTAAGCTTTCGCGTCCAA 3’
HDAC2 FP	5’ AGACTGTCCAGTGTTCGATGGA 3’
HDAC2 RP	5’ CGGCCCCAGCAACTGA 3’
HDAC4 FP	5’ CACTGACGCTGCTAGCAATGA 3’
HDAC4 RP	5’ ACGCGGGCAGGATTCA 3’
G9A FP	5’ CCCTTACCGACAACGAGGAA 3’
G9A RP	5’ TCGGCAATAGCGGCACTAC 3’
Set7 FP	5’ CTCCTTTACTCCAAACTGCGTCTAT 3’
Set7 RP	5’ GGTGCGGATGCACTTGATG 3’
GAPDH FP	5’ AGACAGCCGCATCTTCTTGT 3’
GAPDH RP	5’ CTTGCCGTGGGTAGAGTCAT 3’
18S RNA FP	5’ GTAACCCGTTGAACCCCATT 3’
18S RNA RP	5’ CCATCCAATCGGTAGTAGCG 3’
RPL-19 FP	5’ AATCGCCAATGCCAACTCTCG 3’
RPL-19 RP	5’ TGCTCCATGAGAATCCGCTTG 3’

Forward primer (FP), Reverse primer (RP), Methyl forward primer (MF), Methyl reverse primer (MR), Un methyl forward primer (UF), Un methyl reverse primer (UR), Bisulfite sequencing forward primer (BSPFP), Bisulfite sequencing reverse primer (BSPRP), 5’end biotin labelled (5Bio), Sequencing forward primer (seqFP).

### Western blot analysis for protein measurements

Protein levels of PRL, D2R, DNMT1, DNMT3b, HDAC2 and HDAC4 were determined by western blot analysis. Total protein from each pituitary samples were extracted and the concentration was measured by protein assay reagent (Bio rad Labs, CA, USA). About 30ug of total protein was run in 12% SDS PAGE and transferred to PVDF membrane at 30V overnight at 4°C. The membranes were blocked in 5% non-fat dry milk-TBS-0.1% Tween 20 (TBST) at room temp for 2h. The membranes were incubated with primary antibody in the same blocking buffer at 4°C overnight. The primary antibodies used were rabbit anti-rPRL (PRL-S9; 1:300,000, NHPP, Torrance, CA), rabbit polyclonal D2R (H-50)(1:200; cat# sc-9113); mouse anti-actin monoclonal antibody (JLA20; cat# CP01; 1:5000, Calbiochem, Billerica, MA); rabbit polyclonal DNMT1 (H-300) (1:200 cat #sc-20701), mouse monoclonal DNMT3b (2280C3a) (1:200; cat# sc-81252), rabbit polyclonal HDAC2 (H-54) (1:200; cat# sc-7899), rabbit polyclonal HDAC4 (H-92), (1:200; cat# sc-11418), all from Santa Cruz Biotechnology, Santa Cruz, CA), The membranes were washed in TBST and then incubated with corresponding peroxidase conjugated secondary antibody at room temperature for 1h. The membranes were washed in TBST and incubated with ECL reagent and were developed on the film by autoradiography. The protein band intensities were determined by Alpha imager software (Alpha Innotech, San Laendro, CA) and normalized with corresponding actin band intensity.

### D2R methylation measurement by Methylation specific PCR

D2R methylation was performed by SYBR green real time PCR method of methylation specific PCR (MSP) as it was described previously [32, 34]. DNA was extracted from pituitary samples using All in One Purification Kit (Norgen Bioteck, Ontario, Canada). The bisulfite conversion of DNA was performed using EZ DNA methylation kit (Zymo Research, Orange, CA). Primer sequences specific for methylated and unmethylated DNA were used. Rat highly methylated, low methylated control DNA (Epigen Dx; Hopkinton, MA) were also subjected to bisulfite conversion and were used for preparing the standard curve. Real time quantitative PCR was performed at 95C for 5min followed by 40 cycles of 95C for 15 sec, 60C for 30 sec, 72C for 40 sec. Relative quantity of methylated and unmethylated DNA were measured using the standard curve method. DNA methylation was measured as a ratio of methylated verses unmethylated DNA.

### D2R DNA methylation by Pyrosequencing assay

DNA methylation at specific CpG site was confirmed by pyrosequencing assay. Briefly 1ug of genomic DNA was subjected to bisulfite conversion using EZ DNA methylation kit (Zymo Research, Orange, CA). Regions of interest were amplified from bisulfite treated genomic DNA using pyromark PCR kit (Qiagen; Valencia, CA) with forward and biotin labelled reverse primer as per the instructions from the manufacturer. Biotinylated PCR product was mixed with strepatavidin beads and annealed with sequencing primer. Streptavidin bound biotinylated PCR product was captured using a vacuum filtration sample transfer device (Qiagen). Biotinylated PCR products were annealed with sequencing primer. Sequencing was performed using Pyromark Gold Q96 CDT reagents (Qiagen) on a PSQ HS96A model pyrosequencing machine (Qiagen) as per the instructions from the manufacturer. In the pyrosequencing study, we analysed one control C in non CpG background for efficient C→T conversion. The percent C remaining as C in the target CpG was considered % methylation.

### Statistical analysis

Data were analyzed using Prism 5.0 (GraphPad Software). The data shown in the figures are mean ± SEM. The significant differences between different treatment groups were assessed with one-way analysis of variance (ANOVA) with post-hoc analysis using the Newman Keuls posttest. P<0.05 was considered significant.

## Results

### Fetal alcohol exposure increases plasma PRL level and lactotropic cell proliferation in the pituitary gland

**Fig 1:**
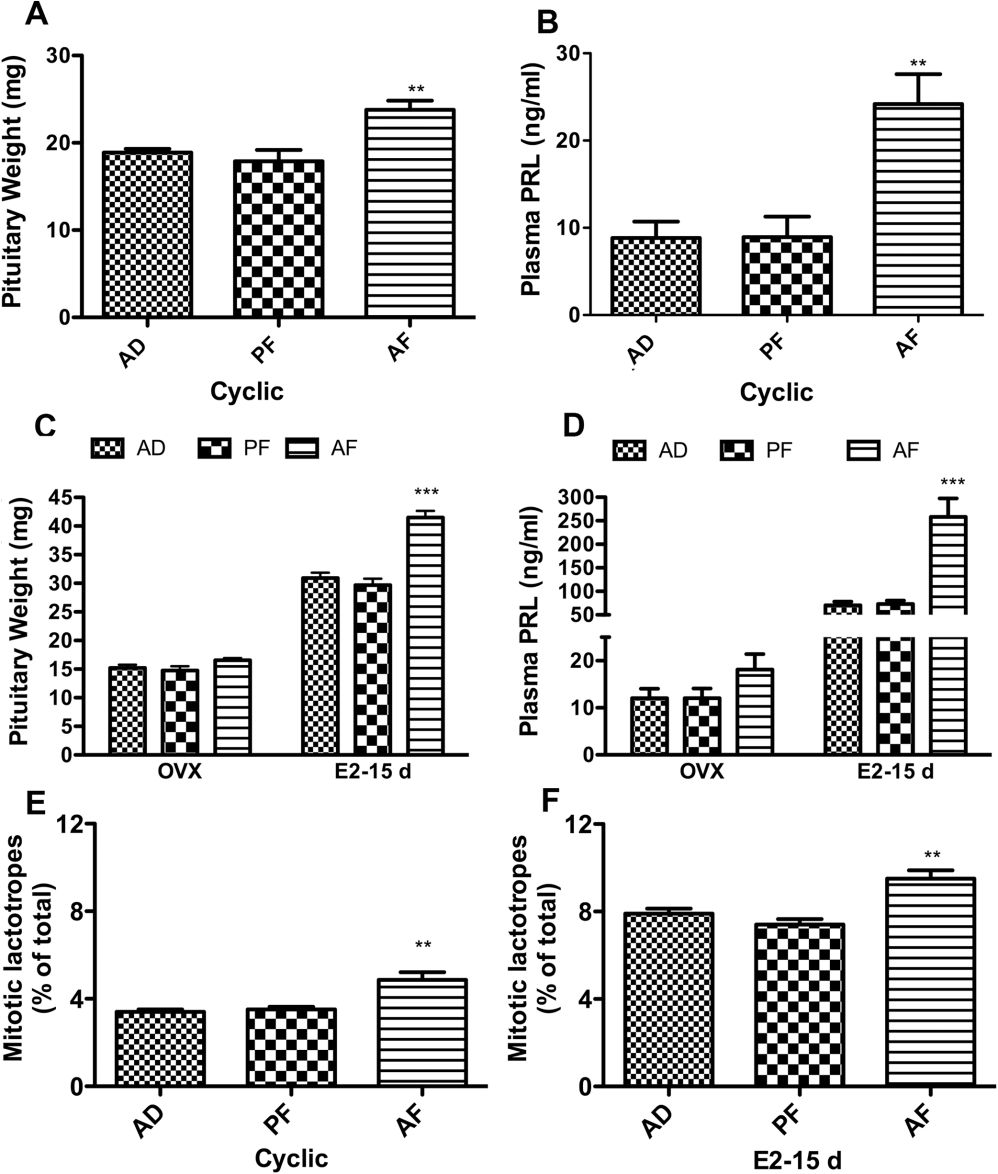
Effects of fetal alcohol exposure on pituitary weight, plasma PRL and percentage of mitotic lactotropes in pituitaries of cyclic, ovariectomized and estrogen-treated ovariectomized animals. Alcohol-fed (AF), pair-fed (PF) or *ad libitum*-fed (AD) rat offsprings were used during the adult period (75–80 days) either on the day of estrus (cyclic) or 15 days after ovariectomy and empty implants (OVEX) or β-estradiol implants (E2-15 d). Mean ± SEM values of pituitary weight (A) and plasma PRL (B) in cyclic animals, pituitary weight (C) and plasma PRL (D) in OVEX or E2-15 d animals, and the percentage of mitotic lactotropes in pituitary of cyclic (E) and E2-15 d (F) animals are shown in the histograms. Data are mean ± SEM of n = 6–8 animals per each group and were analyzed using one-way ANOVA with the Newman-Keuls post hoc test. **, P<0.01 and ***, P<0.001 between AF and controls (AD or PF).

To investigate whether rats that were exposed to alcohol *in utero* display any abnormalities in lactotropic hormone secretion and proliferation during adulthood, cyclic AD, PF and AF rats at three months of age were sacrificed and changes in their pituitary weight and plasma PRL levels were measured. Data shown in Fig 1a and 1B indicate that both pituitary weight and plasma level of PRL were significantly elevated in AF rats as compared to AD and PF controls.

Since estradiol is a physiological regulator of lactotropic cell growth and PRL secretion [29] and since estradiol is known to be produced at a higher level in fetal alcohol exposed rats [11, 12], we evaluated changes in pituitary weight and plasma PRL levels in fetal alcohol exposed rats following ovariectomy and after estrogen replacement for 15 days period. Data shown in Fig 1C and 1D indicate that prenatal ethanol-induced changes in pituitary growth and PRL release are reduced following the steroid removal by ovariectomy and enhanced following the steroid replacement.

To evaluate if the pituitary growth increase in prenatal ethanol exposed rats is caused by enhanced lactotrpic cell proliferation, we determined the percent changes in the number of mitotic lactotrpic cells in the pituitary. Lactotropic cell proliferation in non-estrogen-treated ovariectomized rats was very low (1.8 to 2.1% of total, N = 5). However, cyclic rats at estrus stage and ovariectomized rats after estradiol treatment for 15 days showed detectable number of mitotic lactotropes in the pituitary. These data are shown in Fig 1E and 1F, which demonstrate that prenatal ethanol exposure increased the number of mitotic lactotrpes in the pituitary of both cyclic and estrogen-treated rat offspring. Together these data suggest that fetal alcohol exposure increases plasma PRL level, lactotropic cell proliferation and pituitary growth.

### Fetal alcohol exposure increases estrogen-induced mitogenic action in the pituitary gland

**Fig 2:**
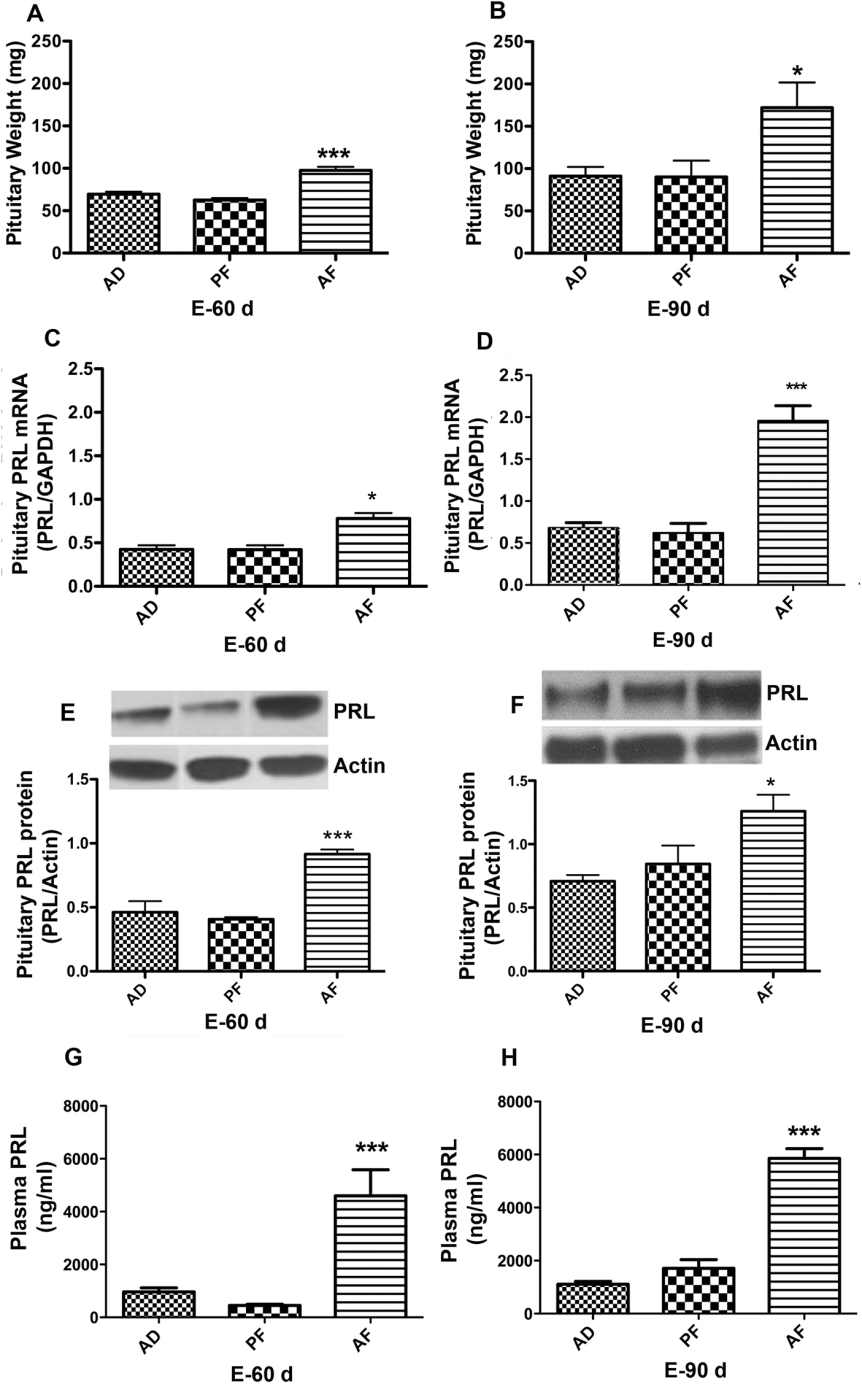
Effect of fetal alcohol exposure on the sensitivity of pituitary lactotropes to the estrogen tumor-promoting action. Alcohol-fed (AF) or control-fed (PF, AD) rats offspring were ovariectomized and implanted with a β-estradiol implants at 60 days of age and after 60 (E2-60 d) or 90 days (E2-90 d) were used for this study. Mean ± SEM values of pituitary weight at 60 d (A) and 90 d (B), pituitary PRL mRNA level at 60 d (C) and 90 d (D), pituitary PRL protein level at 60 d (E) and 90 d (F), and plasma PRL levels at 60 d (G) and 90 d (H) are shown in the histograms. Data are mean ± SEM of n = 4–8 animals per group and were analyzed using one-way ANOVA with the Newman-Keuls post hoc test. *, P<0.05 and ***, P<0.001 between AF and controls (AD or PF).

To further characterize fetal alcohol-induced changes in lactotropic cell function, we evaluated effects of long-term estradiol treatment (60 and 90 days exposure), known to induce prolactinomas in F344 rats [29, 36], on the weight of pituitary, the production of pituitary PRL and the level of plasma PRL in fetal alcohol exposed and control rats. As expected, data shown in Fig 2 indicate that estradiol treatment markedly increased the growth and production of PRL (mRNA and protein levels) in the pituitary gland and PRL levels in plasma in a time-dependent fashion (compare also data shown in Fig 1A–1D). We measured the expression of different internal control genes such as GAPDH, 18S RNA and RPL-19 in pituitary tissue samples to rule out the possible gene expression changes with fetal alcohol exposure. The expression of these internal control genes did not significantly change with the prenatal alcohol exposure (S1 Fig). Interestingly, prenatal ethanol exposures promoted estradiol stimulated growth as well as PRL production and secretion in the pituitary gland. These results suggest the possibility that prenatal ethanol exposure may promote development and or increase the growth of prolactinomas in the pituitary gland.

### Fetal alcohol exposure reduces D2R expression in the pituitary gland

**Fig 3:**
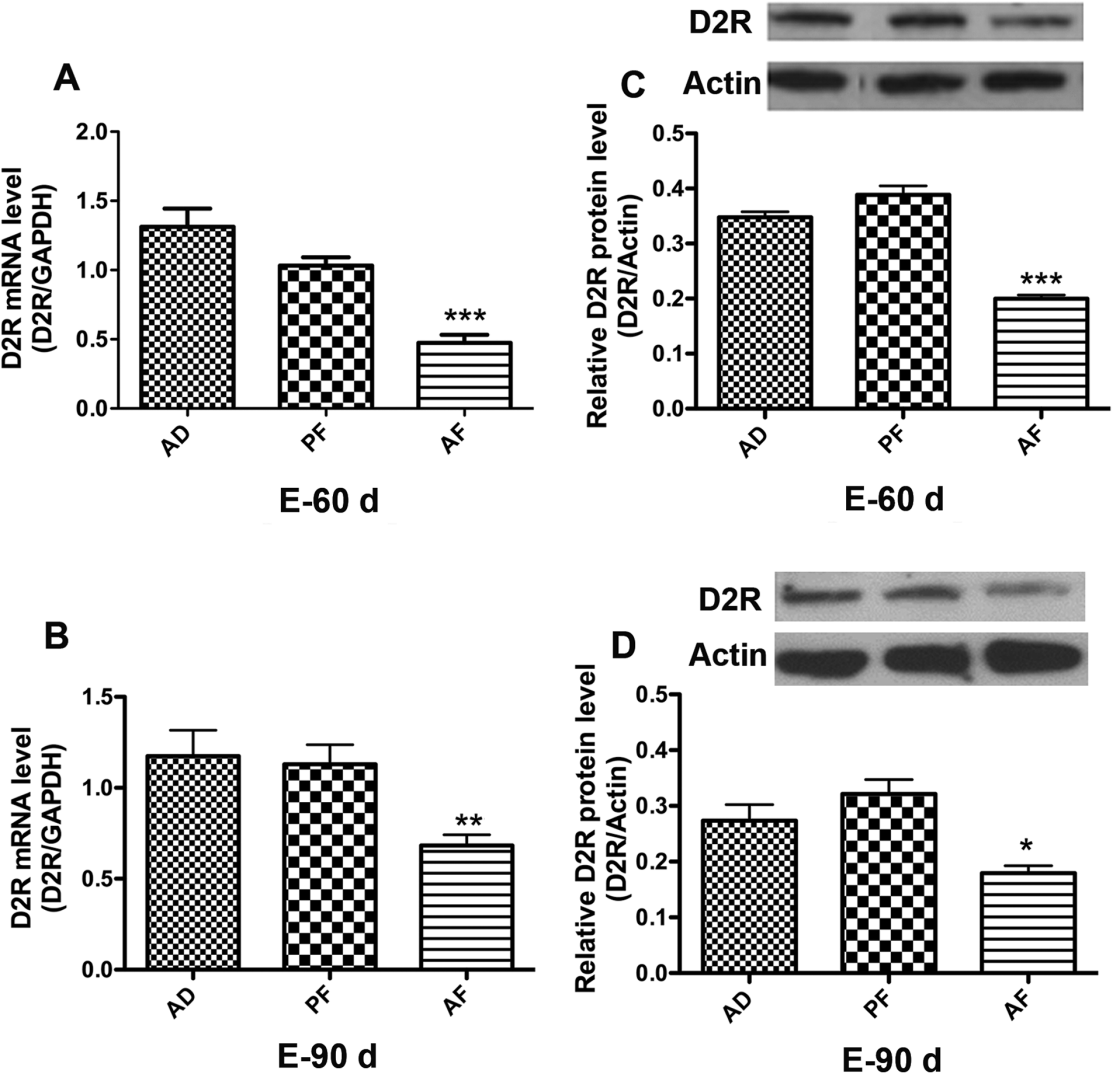
Effect of fetal alcohol exposure on D2R expression in the pituitary. D2R mRNA (A, B) or D2R protein levels (C, D) in pituitaries of AD, PF, AF rat offspring were measured after ovariectomy and 60 or 90 d of estrogen implants, respectively. Data are mean ± SEM of n = 6 animals per each group and were analyzed using one-way ANOVA with the Newman-Keuls post hoc test. *, P<0.05, **, P<0.01, and ***, P<0.001 between AF and controls (AD or PF).

Lactotropic cell proliferation and PRL production in the pituitary glands are controlled by dopamine secreted from the hypothalamus [19–21]. Dopamine agonists are used for treatment of prolactinomas and D2R functional loss or lowered levels of D2R are responsible for resistance to DA agonists [23]. Since we observed that prenatal ethanol exposure increases lactotropic cell proliferation and PRL production in the pituitary, we next sought to determine the level of D2R expression as a measure of dopamine control mechanism. We evaluated D2R expression both at the mRNA and protein level. We found that D2R mRNA expression was significantly reduced in pituitaries of AF rats compared to AD and PF controls both at 60D and 90D after estrogen treatment (Fig 3A and 3B). We also found that the D2R protein level was also significantly reduced in AF pituitaries compared to control groups at 60D and 90D after estrogen treatment (Fig 3C and 3D). These results demonstrate that prenatal ethanol exposure significantly reduces D2R expression in the pituitary gland.

### Fetal alcohol exposure increases CpG methylations of D2R gene promoter in the pituitary gland

**Fig 4:**
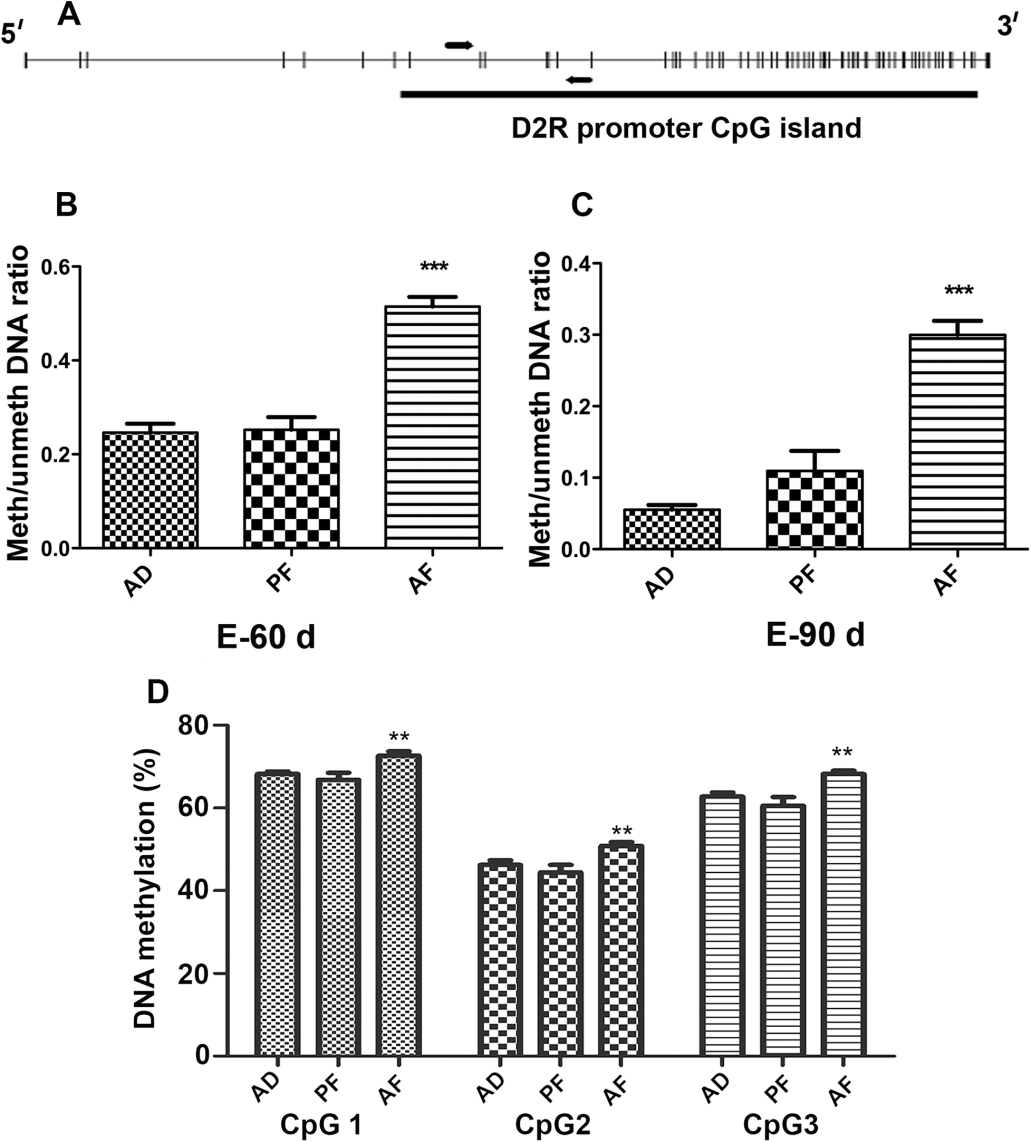
Effect of fetal alcohol exposure on D2R promoter methylation in the pituitary. A. Schematic representation of rat D2R promoter CpG island. Rat D2R promoter sequence was analyzed to search for CpG islands by Methyl primer v1 program. Each small vertical line represents single CpG. The thick solid bar below represents a CpG island. Arrows represent primer annealing sites (A). D2R promoter methylation in pituitaries of AD, PF, AF rats after ovariectomized and estrogen treatment for 60 or 90 days was performed by real time methylation specific PCR. Methylation was measured as ratio of methylated verses unmethylated DNA. D2R methylation in pituitary of AD, PF, AF rats after ovariectomized and estrogen treatment for 60 (B) and 90 days (C). Pyrosequencing analysis of 3 individual CpGs in the CpG island of the D2R promoter in pituitaries of AD, PF, AF rats after ovariectomized and estrogen treated for 60 days (D). Data are mean ± SEM of n = 6 animals per each group and were analyzed using one-way ANOVA with the Newman-Keuls post hoc test. **, P<0.01 between AF and control AD or PF.

Because fetal alcohol exposure produces long-lasting changes in D2R expression in the pituitary gland, the possibility arose that an epigenetic signature by alcohol may be altering the D2R promoter activity in the offspring. CpG island methylation in the gene promoter is one of the epigenetic regulations of gene expression. We analyzed the promoter sequences of the rat D2R using the Methyl primer express v1.0 software (ABI) and found a large CpG island extended upstream of the transcriptional start site (Fig 4A). Promoter DNA methylation determined by Methylation specific PCR (MSP) assay revealed that D2R promoter DNA methylation was increased in the pituitary of AF rats compared to AD and PF controls at 60D and 90D after estrogen treatment (Fig 4B and 4C). We further confirmed the methylation status of single CpG sites using the pyrosequencing assay from the samples obtained after 60 days of estrogen treatment. We analyzed three independent CpG sites in the D2R promoter and found that the percent of methylation is increased in AF compared to AD and PF rat offspring (Fig 4D). These results suggest that fetal alcohol exposure makes an epigenetic mark involving hypermethylation of the D2R promoter in the pituitary gland.

### Fetal alcohol exposure increases the activity of genes controlling DNA methylation and histone modification associated with transcriptional repression in the pituitary gland

**Fig 5:**
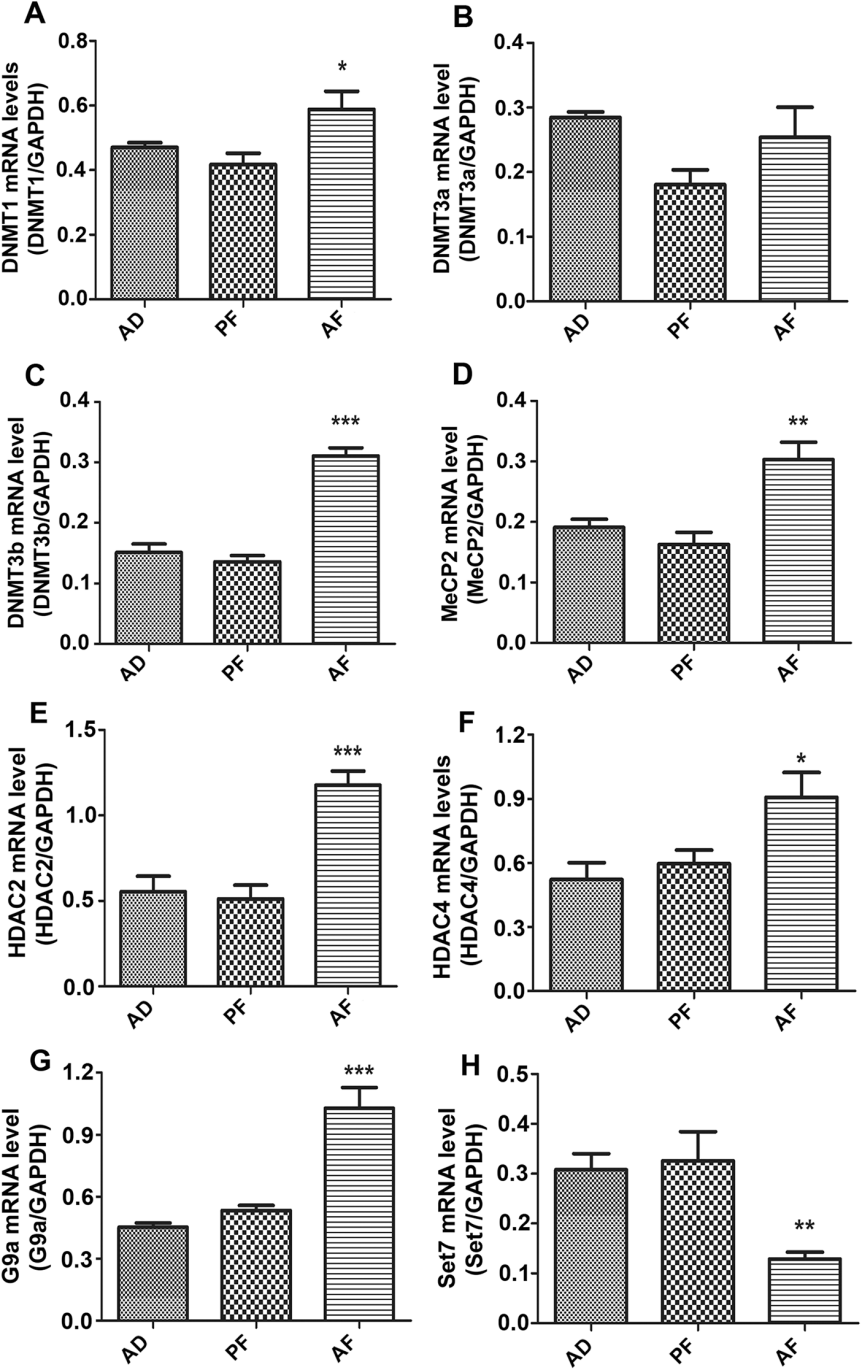
Effect of fetal alcohol exposure on the level of genes regulating DNA methylation and histone modification in the pituitary. Pituitary mRNA levels of DNMT1 (A), DNMT3a (B), DNMT3b (C), MeCP2 (D), HDAC2 (E), HDAC4 (F), G91 (G) and Set7 (H) of AD, PF. AF rats after ovariectomy and estrogen treatment for 60 days. Data are mean ± SEM of n = 6 animals per group and were analyzed using one-way ANOVA with the Newman-Keuls post hoc test. *, P<0.05, **, P<0.01, and ***, P<0.001 between AF and controls (AD or PF).

**Fig 6:**
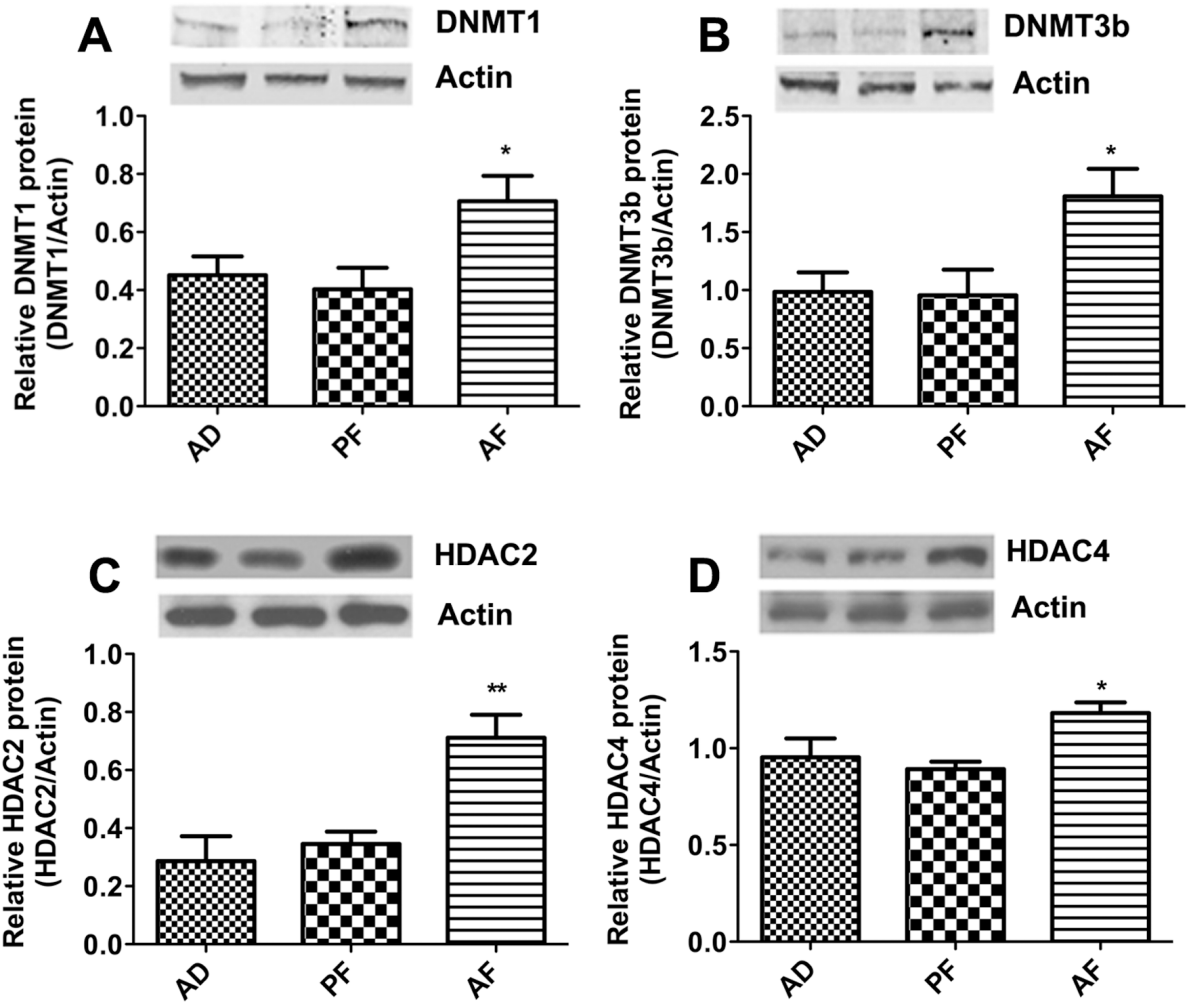
Effect of fetal alcohol exposure on DNA methylation and histone deacetylation proteins in the pituitary. Pituitary protein levels of DNMT1 (A), DNMT3b (B), HDAC2 (C), HDAC4 (D) of AD, PF and AF rats after ovariectomy and estrogen treatment for 60 days. Data are mean + SEM of n = 6 animals per group and were analyzed using one-way ANOVA with the Newman-Keuls post hoc test. *, P<0.05, **, P<0.01 between AF and controls (AD or PF).

DNA methylation is carried out by addition of a methyl group at the cytosine residue of CpG dinucleotides by utilizing S-adenosyl methionine as a substrate catalyzed by DNA methyl transferases [37]. DNMT1, DNMT3A and DNMT3B are the important DNA methyl transferases. Methyl CpG binding protein2 (MeCP2) binds to hypermethylated promoter and represses the transcription. Histone deacetylases are enzymes involved in covalent histone modification resulting in condensed chromatin conformation and decreased gene expression [38, 39]. HDAC2 and 4 are the Class I, II HDACs that catalyze deacetylation of histones thereby repressing gene transcription. G9A is a histone methyl transferase that catalyzes H3K9 and H3K27 methylation involved in transcriptional repression, while SET7 is a H3K4 methyl transferase associated with positive transcription. Determination of these gene expression levels in the pituitary following 60 days of estrogen treatment in fetal alcohol exposed and control rats revealed that the levels of DNMT1 and DNMT3B, but not DNMT3A, were significantly increased in AF rats compared to AD and PF controls (Fig 5A, 5B and 5C). Fetal alcohol exposure also increased the expression of MeCP2 (Fig 5D) as well as HDAC2 and HDAC4 in AF rat pituitaries as compared to AD and PF rat pituitaries (Fig 5E and 5F). G9A expression was also increased, while SET7 expression was reduced in pituitaries of fetal alcohol exposed rat offspring (Fig 5G and 5H). We also confirmed the expression of DNMT1 and 3b proteins essential for DNA methylation, HDAC2 and HDAC4 proteins essential for histone deacetylation by western blot analysis. Our results revealed that the expression of these proteins significantly increased in AF rat pituitaries compared to AD and PF controls after 60 days of estrogen treatment (Fig 6). These results suggest that fetal alcohol exposure increases expression of various epigenetic modifier genes associated with transcriptional repression.

### Fetal alcohol induced changes in D2R expression and lactotropic function is reversed in the pituitary gland by epigenetic modifying drugs

**Fig 7:**
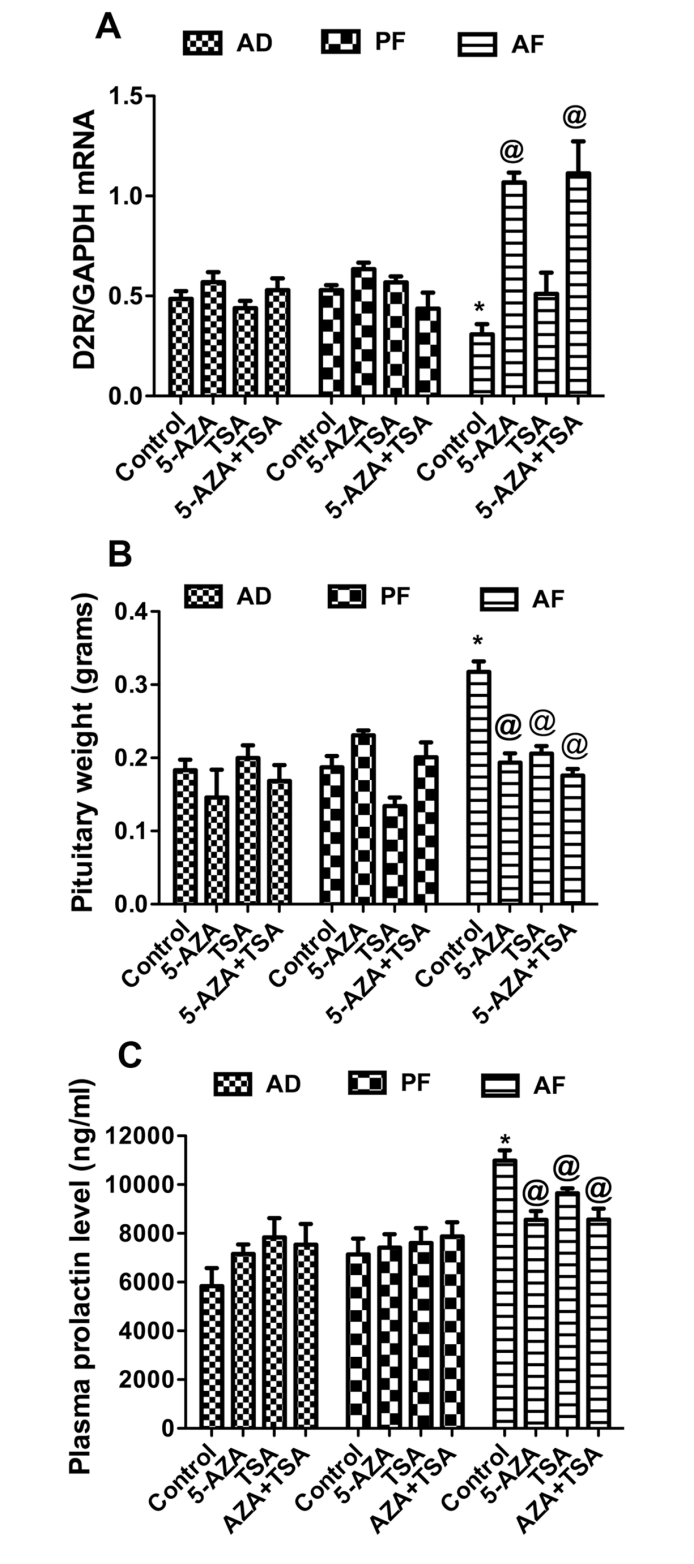
Effects of epigenetic modulatory drugs on fetal alcohol induced changes in pituitary D2R mRNA levels, protein levels, pituitary weight and plasma PRL levels. AD, PF and AF rat offspring were treated with DNA methyl transferase inhibitor 5-Aza deoxycytidine (5-AZAdC), HDACC inhibitor Trichostatin-A (TSA) alone or together during postnatal day 2–6 and after 60 days, these rats were ovariectomized and estrogen treated for 60 days. Mean ± SEM values of pituitary D2R mRNA levels (A), pituitary weight (B) and plasma PRL levels (C) are shown in the histograms. Data are mean ± SEM of n = 6 animals per group and were analyzed using one-way ANOVA with the Newman-Keuls post hoc test. *, P<0.05, between AF and controls (AD or PF). @, P<0.05 between the treatment and control in AF group.

In order to establish the functional role of epigenetic modifications associated with fetal alcohol exposure in repression of D2R expression and prolactinoma development, we tested the effects of epigenetic modulatory drugs such as 5-Aza-dC (a DNA methylation inhibitor), TSA (a HDAC inhibitor) and the two drugs in combination on the estradiol-induced changes in the levels of pituitary D2R mRNA, pituitary weight and plasma PRL. We found that 5-Aza-dC significantly increased D2R expression in AF animals compared to the vehicle control (Fig 7A). TSA treatment moderately increased D2R expression in AF compared to vehicle control, but it did not achieve significance. 5-AZAdC and TSA in combination also significantly increased D2R expression compared to vehicle control. We also found that 5-Aza-dC treatment significantly increased pituitary D2R protein levels compared to vehicle control after 60 days of estrogen treatment. The increase of D2R protein is higher in AF animals compared AD or PF controls (S2 Fig). We found that both 5-Aza-dC and TSA were effective in reducing pituitary weight and plasma PRL levels in AF rats to the extent that these values are not different from AD and PF controls (Fig 7B and 7C). These drugs did not change pituitary weight and plasma PRL levels in AD and PF rats, suggesting that the epigenetic modulatory drugs reverse fetal alcohol induced epigenetic modifications thereby increasing D2R expression that results in reduced pituitary weight and plasma PRL levels. Together these data suggest that fetal alcohol exposure epigenetically programs the pituitary to repress D2R gene expression and stimulate pituitary lactotropic cell growth and PRL production.

## Discussion

Epigenetic changes are now being considered as potential mechanisms for the delayed effects of many toxicants when individuals are exposed to them during development [40]. Epigenetic modifications are most commonly regulated by direct methylation of DNA and/or by posttranslational modification of histones, both of which can either promote or repress gene transcription [41, 42]. Epigenetic dysregulation, promoter methylation and silencing of tumor suppressor genes are implicated in pituitary neoplasia [43–48]. Overall, the data presented here indicate that fetal alcohol exposure resulted in hypermethylation of D2R gene promoter in adulthood, even though the exposure to ethanol ended at GD 21. Although chronic alcohol consumption causes hypomethylation in general, it’s effect on DNA methylation is tissue specific and depend on specific gene targets and developmental stage of exposure. Prenatal alcohol exposure causes DNA hypermethylation and results in reduced expression of brain derived neurotrophic factor (BDNF) in olfactory bulbs associated with the loss of neurons in this brain region [49]. It has also been reported in neural cell culture, alcohol increases DNMT activity and hypermethylation of promoters of cell cycle regulated genes causing down regulation of these genes [50]. Previously it has been shown that fetal alcohol exposure significantly affects D2R expression [17] and function [51]. In the present study the results showed that the increase in D2R promoter methylation was correlated with a decrease in D2R mRNA levels and increased PRL mRNA levels in the pituitary of AF rats. Since ethanol is known to suppress D2R expression [17], it could be hypothesized that fetal alcohol exposure suppresses D2R expression through hypermethylation of the gene promoter.

Pituitary tumorogenesis has shown to often involve genetic mutations of classical oncogenes or tumor suppressor genes [52, 53]. Epigenetic dysregulation, promoter methylation and silencing of tumor suppressor genes are also implicated in pituitary neoplasia [43–48]. We presented here evidence to show that fetal alcohol exposure hypermethylates D2R promoter and reduces its mRNA and protein productions. D2R protein plays an important role in regulation of growth and secretion of PRL in pituitary lactotropes [20–22, 29]. Literature suggests that knocking down the D2R gene induces hyperplasia in the pituitary lactotropes in mice [24]. Also evidence is presented to show that high proliferating GH3 cells demonstrate lower D2R mRNA and higher D2R gene methylation than those in low proliferating MMQ cells and normal pituitary gland [54]. These authors also reported that histone code modifications at the D2R promoter in GH3 and MMQ cells correlate with D2R gene expression. We also showed that fetal alcohol exposure increased expression of DNMT1, 3B, MeCP2, HDAC2, 4 and G9A which correlated with increased D2R promoter methylation and the reduced expression of D2R in rat pituitary. DNMT1 is a maintenance methyl transferase, DNMT3A and 3B are de novo methyl transferases. DNMT3B has been reported to be over expressed in pituitary tumors [55]. We previously reported that the increased DNA methyl transferases results in hypermethylation of POMC promoter and reduced expression in the hypothalamus of fetal alcohol exposed rat offspring [31]. We also reported that fetal alcohol exposure increases MeCP2 expression to recruit on to POMC gene promoter thereby repressing the POMC gene transcription in the hypothalamus [33]. All these epigenetic modifiers play a critical role in DNA methylation, which in turn regulate the transcriptional repression. Our results also revealed that fetal alcohol exposure increased HDAC2, 4, G9A and reduced the expression of Set7. These histone code modifying enzymes play important role in histone deacetylation and methylation. HDAC2 and 4 are class I and II HDAC family members and known to play a role in transcriptional repression. G9A is a euchromatic histone methyl transferase catalyzes H3K9 and H3K27 methylation correlate with negative transcription. Set7 mediated H3K4 methylation correlates with positive transcription. The data of the present study indicate that FAE induced histone code modifications were correlated to transcriptional repression suggesting their role in reduced D2R expression. There is a complex interplay between DNA methylation and histone code modifications, the two epigenetic layers to regulate gene expression. The role of DNA methylation and histone deacetylation in D2R repression of FAE rat pituitaries were further confirmed in the study where we determined the effects of DNA methylation inhibitor 5-Aza-dC and HDAC inhibitor TSA. We found that 5-AzaDC treatment increased D2R expression in AF rat offspring (Fig 7). We also observed a marked increase in D2R protein levels in AF rats and only a moderate increase in AD rat offsprings (S2 Fig). 5-Aza-dC is a cytosine analogue incorporated into DNA during DNA replication thereby it traps the DNMT1 and inactivate its function. Hence, the 5-Aza-dC effect on D2R expression in AF rats might be a result of its inhibitory action on DNMT1-mediated DNA methylation of D2R gene. TSA treatment did not show a significant increase in D2R expression. Both the drugs combination increased D2R expression but did not show any additive effect. Plasma PRL levels and pituitary weights were significantly reduced in AF rats with the 5-AzadC treatment, and these changes correlated well with the increased D2R expression observed after 5-AzadC treatment in this treatment group. TSA also reduced the plasma PRL level and pituitary weight in AF rats without affecting the D2R level in these rats. These effects of TSA on PRL secretion and pituitary weight may be related to non-D2R-dependent mechanism and need further investigation. Hence, our studies unmasked the epigenetic mechanisms associated with a reduced expression of D2R in prenatal alcohol exposed rat offspring and provide a pharmacological avenue for reactivation of the silenced D2R and suppression of tumor growth using epigenetic modulatory drugs such as 5-AZAdC and TSA. Our study may have relevance for women drinking alcohol during pregnancy who may be increasing their offspring risk for developing prolactinomas. More studies are needed to understand fetal alcohol programming of pituitary for developing pituitary tumors in human.

**Fig 8:**
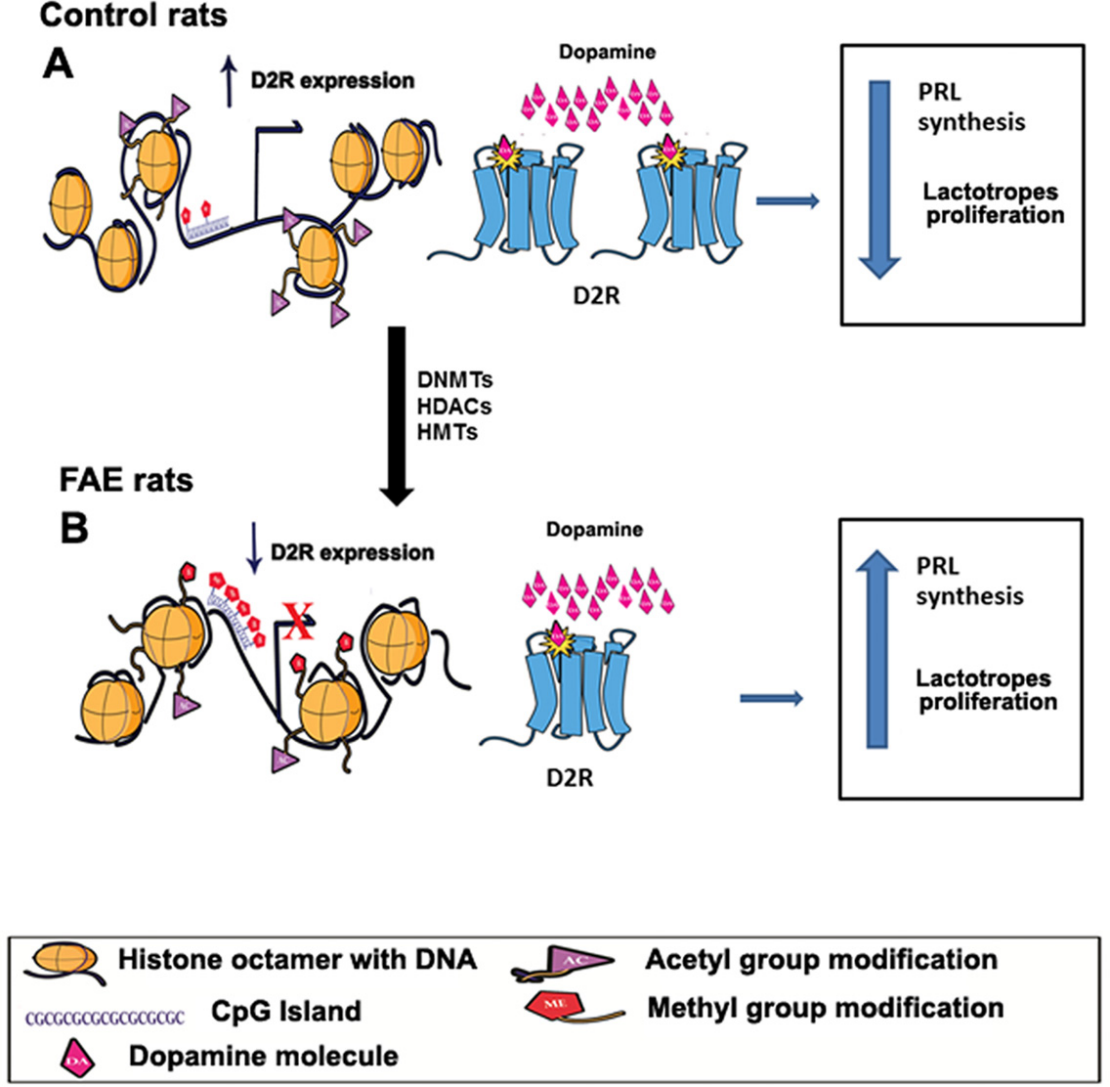
Schematic diagram illustrating fetal alcohol exposure induced epigenetic changes regulating D2R expression and its control of PRL synthesis and lactotropic cell growth in the pituitary. In control rats, D2R mRNA expression is regulated by the epigenetic mechanism involving DNA demethylation and histone deacetylation of the D2R promoter. D2R participates in mediating the inhibitory action of dopamine on PRL synthesis and cell proliferation in lactotropic cells of the pituitary gland (A). In fetal alcohol exposed (FAE) rats, increased D2R promoter methylation and histone deacetylation result in reduced D2R expression, The lower number of D2Rs prevents dopamine to act on lactotropes causing more PRL production and increased cell proliferation (B). DNA wrapped with histone octomer is represented as black thread with cylindrical structures. Methylated CpG is represented as pentagon structure in the DNA. Acetyl groups of histones are represented as triangles and methyl groups as pentagons on N terminal tails of histone.

In conclusion the present study demonstrates that fetal alcohol exposure programs the pituitary to increase susceptibility to tumor development by possibly enhancing the promoter hypermethylation and causing the repression of the D2R gene and its cell growth inhibitory function in the pituitary gland (Fig 8).

## Supporting Information

S1 Fig (PDF)

S2 Fig (PDF)
