# Supplementary figures and images for: Fetal Alcohol Exposure Reduces Dopamine Receptor D2 and Increases Pituitary Weight and Prolactin Production via Epigenetic Mechanisms
Source: PLoS One. 2015 Oct 28;10(10):e0140699. doi: 10.1371/journal.pone.0140699 (PMC4624904; doi:10.1371/journal.pone.0140699)

A

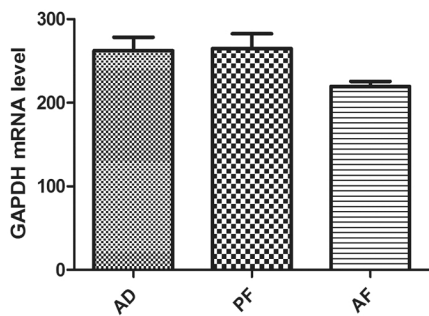

B

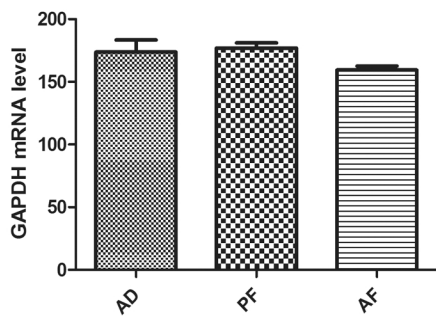

C

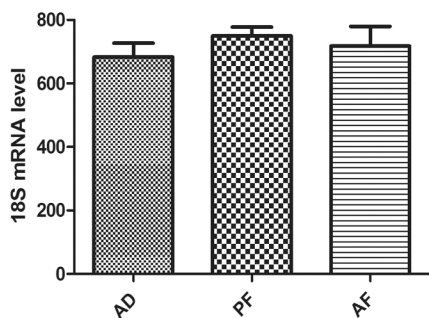

D

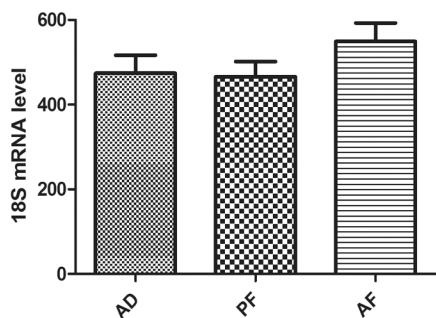

E

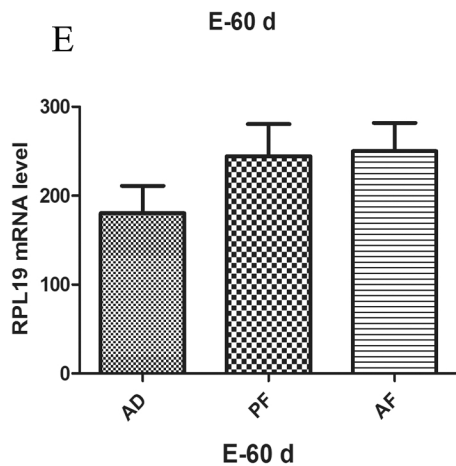

F

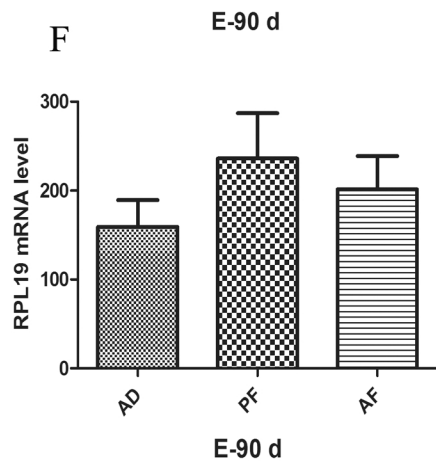

Supplement: S1 Fig — (PDF) [file pone.0140699.s001.pdf]

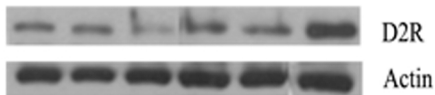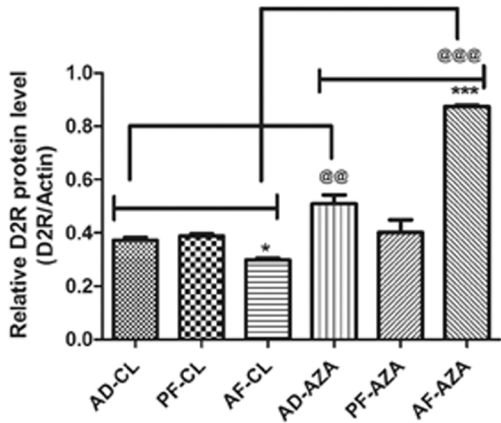

Supplement: S2 Fig — (PDF) [file pone.0140699.s002.pdf]
